# *Lippia origanoides* Essential Oil or Thymol in Combination with Fluconazole Produces Damage to Cells and Reverses the Azole-Resistant Phenotype of a *Candida tropicalis* Strain

**DOI:** 10.3390/jof9090888

**Published:** 2023-08-30

**Authors:** Carolina Zapata-Zapata, Mauricio Rojas-López, Liliana T. García, Wendy Quintero, María C. Terrón, Daniel Luque, Ana C. Mesa-Arango

**Affiliations:** 1 Academic Group of Epidemiology, Faculty of Medicine, University of Antioquia, Medellín 050010, Colombia; carolina.zapataz@udea.edu.co; 2 Group of Cellular Immunology and Immunogenetics (GICIG), Faculty of Medicine, University of Antioquia, Medellín 050010, Colombia; mauricio.rojas@udea.edu.co; 3 Flow Cytometry Unit, University of Antioquia, Medellín 050010, Colombia; 4 Postgraduate Department of Infectious Disease, University of Santander, Bucaramanga 680006, Colombia; l.torcoroma@udes.edu.co (L.T.G.); wendy.quinterog@udea.edu.co (W.Q.); 5 Electron Microscopy Unit, Scientific-Technical Central Units, Institute of Health Carlos III (ISCIII), 28220 Madrid, Spain; mcterron@isciii.es (M.C.T.); dluque@isciii.es (D.L.)

**Keywords:** essential oil, *Lippia origanoides*, antifungal activity, synergism, *Candida tropicalis*

## Abstract

*Candida tropicalis* is one of the most pathogenic species within the genus. Increased antifungal resistance has been reported, which is in part due to the organism’s ability to form biofilms. In natural products derived from plants, such as essential oils (EOs) or their major components, there is significant potential to develop new antifungals or to both enhance the efficacy and reduce the toxicity of conventional antifungals. This study aimed to evaluate the effect of combining an EO of *Lippia origanoides* or thymol with fluconazole on an azole-resistant *C. tropicalis* strain. Synergism was observed in the combination of fluconazole with the EO and with thymol, and minimal inhibitory concentrations for fluconazole decreased at least 32-fold. As a consequence of the synergistic interactions, mitochondrial membrane potential was reduced, and mitochondrial superoxide production increased. Alteration in nuclear morphology, cell surface, and ultrastructure was also observed. In conclusion, the synergistic interaction between *L. origanoides* EO or thymol with fluconazole reverted the azole-resistant *C. tropicalis* phenotype. These findings suggest that *L. origanoides* EO or thymol alone, or in combination with fluconazole, have the potential for development as antifungal therapies for this yeast, including resistant strains.

## 1. Introduction

*Candida* species are opportunistic pathogens that cause infections with a wide range of clinical presentations. These infections have increased in recent years, particularly in immunocompromised patients [1]. Although *Candida albicans* remains the most prevalent species worldwide, non-*albicans* species such as *C. tropicalis*, C*. parapsilosis*, *C. glabrata*, *C. krusei*, and *C. auris* are now recognized as relevant pathogens [2].

*Candida tropicalis* is a constituent of the human microbiome and is the cause of a number of common infections, including onychomycosis and oral, genital, and skin candidiasis [3,4]. More seriously it can cause invasive infections associated with high morbidity and mortality [5,6]. As a consequence, this yeast is included in the list of fungal priority pathogens by the World Health Organization (WHO) [7].

Studies carried out in Latin America have shown that *C. tropicalis* is one of the more common species identified in cases of candidemia [2,8]. In Colombia, *C. tropicalis* is the second-most isolated species from patients with bloodstream infections [9].

Current therapies for *Candida* species infections are based mainly on azoles, amphotericin B (AMB), and echinocandins [10]. However, the resistance in different species of *Candida* to these antifungals has been documented in the scientific literature. Although, in general, *C. tropicalis* is susceptible to antifungals, several studies have demonstrated the emergence of clinically resistant isolates of *C. tropicalis* to one or various classic antifungals [11,12,13]. Furthermore, the use of these antifungals is limited by their toxicity and low selectivity [14,15,16].

Natural products are a well-documented source of molecules with biological activities [17]. As a result, different bioactive molecules have been developed, such as the anti-cancer vinca alkaloids and the paclitaxel terpene derived from the *Catharanthus roseus* and *Taxus brevifolia* plants, respectively. The antimalarial artemisinin (sesquiterpene lactone) from *Misia annua* L and the blood cholesterol reducer lovastatin (statin) obtained from *Aspergillus terreus* are further examples [18,19].

Currently, essential oils (EOs) distilled from different plants around the world are of interest in the search for new antifungal compounds with targets or mechanisms of action different from those of conventional antifungals for clinical use, or that have a synergistic effect in combination with known antifungals [20,21]. Relevant activity against different *Candida* species has been identified in EOs from aromatic plants such as *Melaleuca alternifolia* L., *Thymus vulgaris* L., *Mentha piperita* L., *Rosmarinus office*, *Juniperus oxycedrus* L., *Cinnamomum zeylanicum*, and *Ruta graveolens* [22,23,24,25,26,27]. The anti-*Candida* spp. activity of EOs and terpenes has been related to the alteration in cell membrane permeability, DNA integrity, the calcium signaling pathway, cell cycles, mitochondrial functions, and an increase in the level of intracellular reactive oxygen species (ROS) [28,29,30].

Colombia is a megadiverse country that ranks in fourth place internationally in abundance and diversity of plant species [31,32]. This may provide an opportunity to discover EOs with antifungal activity. The aromatic species of plants of the *Lippia* (Verbenaceae) genus, have been studied in Colombia for their biological activities [33,34,35]. *Lippia origanoides* is a species of aromatic plant commonly known as “mountain oregano” and has been used by native communities for its medicinal properties [36,37]. Phytochemical analysis of *L. origanoides* EOs indicates that they are composed mainly of terpenes, terpenoids, and phenylpropanoids. According to this analysis, five *L. origanoides* chemotypes have been described: *p*-cymene, *α*- and *β*-phellandrene, and limonene (chemotype A); carvacrol (chemotype B); thymol (chemotype C); 1,8-cineole (chemotype D); and (E)-methyl cinnamate and (E)-nerolidol (chemotype E) [38]. In a previous study carried out by our group, the activity of EOs derived from different chemotypes of *L. origanoides* against *Candida* spp. with different antifungal sensitivity profiles was demonstrated [39].

Combination therapy is a well-recognized approach to overcoming fungal resistance and maximizing the therapeutic efficacy and selectivity of antifungals [40,41]. The synergistic effect of fluconazole (FLC) has been demonstrated with other antifungals of clinical use and other agents with specific activity against *Candida* spp. [42,43,44]. EO and terpenes combined with FLC have been shown to be effective against FLC-resistant *Candida* spp. [45].

In this study, we focused on evaluating the effect of the interaction of an EO distilled from the *L. origanoides* (thymol + *p*-cymene) chemotype or of thymol in combination with FLC against an azole-resistant *C. tropicalis* strain on specific cell functions and structures.

## 2. Materials and Methods

*Candida tropicalis* ATCC 200956 and *C. tropicalis* ATCC 750 strains, resistant and susceptible to azoles, respectively, were obtained from the American Type Culture Collection (ATCC, Manassas, VA, USA) and cultured on Sabouraud Dextrose Agar (SDA; Sigma-Aldrich, St. Louis, MO, USA). The *Candida tropicalis* ATCC 200956 strain carries a 132 bp deletion in the *ERG11* gene (44 amino acids) and a C773T substitution in the *ERG3* gene, leading to the S258F mutation. The aforementioned changes provide this strain with cross-resistance to both azoles and AMB [11].

### 2.1. Compounds

The EO of the *L. origanoides* (thymol + *p*-cymene) chemotype was obtained via hydrodistillation and characterized by gas chromatography-mass spectrometry (GC-MS), as previously described by Zapata-Zapata et al. [39] and as shown in Table 1.

Thymol (2-isopropyl-5-methyl phenol) and FLC were obtained commercially (Sigma-Aldrich, St. Louis, MO, USA). Stock solutions of compounds were prepared in dimethyl sulfoxide (DMSO; Sigma-Aldrich, St. Louis, MO, USA). Following this, work solutions were prepared using RPMI (Roswell Park Memorial Institute 1640) medium (Sigma-Aldrich, St. Louis, MO, USA) and buffered with 3-(N-morpholino) propane-sulfonic acid (MOPS) (Sigma-Aldrich, St. Louis, MO, USA).

### 2.2. Minimum Inhibitory Concentration

The minimum inhibitory concentration (MIC) of FLC was determined according to the Clinical and Laboratory Standards Institute M27, 4th edition [46]. The antifungal activity of EO and thymol was also evaluated using the same protocol, with some modifications. In brief, work solutions were prepared at 512 µg/mL 2× (two-fold final concentration); then, 100 µL of five serial two-fold dilutions were dispensed in 96-well plates (Corning^®^, Costar^®^, New York, NY, USA), thus achieving final concentrations between 16 and 256 µg/mL after the addition of the same volume of inoculum at a concentration of 2.5 × 10^3^ CFU/mL (2×). Microdilution plates were incubated at 35 °C for 24 h. MICs were visually determined at the lowest concentration that produced visual inhibition compared to the growth control (inoculum without compounds). The assays were performed at least three times in duplicate on different days. The data were expressed as geometric media (GM) of the MIC values (GM-MICs). As antifungal susceptibility testing control, the activity of itraconazole and AMB (Sigma-Aldrich, St. Louis, MO, USA) against the reference strains *C. krusei* ATCC 6258 and *C. parapsilosis* ATCC 22019 was evaluated.

### 2.3. Interaction Assay

Interaction between the EO or thymol with FLC was evaluated against a *C. tropicalis*-resistant strain as well as in the susceptible one, following the fixed ratio method described by Fivelman et al. [47]. Initially, concentrations equivalent to eight-fold (8×) MIC of the FLC, EO, or thymol were prepared as indicated in Table 2.

A total of 100 µL was taken from each combination and dispensed in 96-well plates, and six serial two-fold dilutions were prepared from each combination. Subsequently, 100 µL of the previously prepared suspension of *C. tropicalis* adjusted to 2.5 × 10^3^ CFU/mL (2×) in RPMI + MOPS was added to each well, for a final volume of 200 µL per well. Then, the plates were incubated at 35 °C for 24 h in a humid chamber. MIC values of EO/FLC or thymol/FLC mixes were determined as described above. The assays were performed in triplicate in three independent experiments. MIC values were used to calculate the fractional inhibitory concentration (FIC) using the following formula [48]:

$$\mathrm{FIC}\;\mathrm{compound}\;A=\frac{\mathrm{MIC}\;\left(A\right)\;\mathrm{in}\;\mathrm{combination}\;}{\mathrm{MIC}\;\left(A\right)\;\mathrm{alone}}$$

$$\mathrm{FIC}\;\mathrm{compound}\;B=\frac{\mathrm{MIC}\;\left(B\right)\;\mathrm{in}\;\mathrm{combination}\;}{\mathrm{MIC}\;\left(B\right)\;\mathrm{alone}}$$

The results of the combinations were interpreted by calculating the FIC index (FICI = ∑FIC). Values of FICI ≤ 0.5, 0.5 < FICI ≤ 4, and >4 allowed us to categorize the interactions as synergism, indifference, and antagonism, respectively [49]. Additionally, the FIC values were used to construct isobolograms. The FIC of FLC was plotted on the *x* axis and the FIC of EO or thymol on the *y* axis. An additivity line that joins the theoretical points of the MIC values for each compound (FIC = 1) was drawn. Results were interpreted according to Huang et al. [50]. Experimental points below the additivity line indicated a synergistic effect.

### 2.4. Assessment of Plasmatic Membrane Integrity

The plasma membrane integrity was tested using propidium iodide (PI) (BD^TM^ DNA QC Particles, San José, CA, USA). *Candida tropicalis* ATCC 200956 cells (4 × 10^5^ CFU/mL (2×)) were treated with EO/FLC or thymol/FLC MICs. After incubation at 35 °C for 4 h, cells were stained with PI (2 ng) and incubated at room temperature for 30 min. The percentage of PI-positive cells was measured by a flow cytometer (CitoFlexS™ Beckman Coulter, Indianapolis, IN, USA). The results were analyzed with the FlowJo software, version 7.6 (BD, Franklin Lakes, NJ, USA). Cells heated to 56 °C for 1 h were used as a positive control.

### 2.5. Evaluation of Mitochondrial Membrane Potential (∆ψm)

*Candida tropicalis* ATCC 200956 cells [4 × 10^5^ CFU/mL (2×)] were treated with the previously established MICs for EO/FLC or thymol/FLC and incubated at 35 °C for 4 h to evaluate changes in ∆ψm. Subsequently, cells were stained with 2 µM of 5,5,6,6-tetrachloro-1,1′,3′,3′-tetraethyl benzimidazole carbocyanine iodide MitoProbe^TM^ dye (JC-1) (Invitrogen, Carlsbad, CA, USA) and with 2 ng of 3,3’-Dihexyloxacarbocyanine Iodide [DiOC_6_(3)] (Invitrogen, Carlsbad, CA, USA) and then incubated at 37 °C and at room temperature, respectively, for 30 min in the dark. The cells stained with JC-1 were visualized by fluorescence microscopy (Nikon Eclipse, Tokyo, Japan). Furthermore, the fluorescence of cells stained with JC-1 and DiOC_6_(3) was analyzed using a CitoFlexS™. The data obtained were analyzed by FlowJo 7.6. The red/green ratio was calculated with the data of median fluorescence intensity of cells stained with JC-1, as described by Sivandzade et al. [51]. The percentage of high and low cells for DiOC_6_(3) uptake was also calculated. Both cells untreated and treated with AMB at 4 µg/mL were included as controls.

### 2.6. Mitochondrial Superoxide Indicator

The production of superoxide anion (O_2_-) by *C. tropicalis* ATCC 200956 cells treated with EO/FLC or thymol/FLC was evaluated using MitoSOX^TM^ Red (Invitrogen, Carlsbad, CA, USA). An inoculum of 4 × 10^5^ CFU/mL (2×) prepared using RPMI + MOPS was exposed to MICs at 35 °C for 4 h. Following this, the cells were collected by low-speed centrifugation, washed twice in 1× phosphate-buffered saline (PBS), and stained with MitoSOX^TM^ Red 2 µM at 37 °C for 1 h in the dark. At least 15 cells from each tested treatment were visualized by fluorescence microscopy, and fluorescence intensity was quantified by NIS-Element 4.0.0. software (Tokyo, Japan) according to the manufacturer’s instructions. For comparisons between treatments, the Kruskal–Wallis test was performed using the Prism 6.0 statistical program (GraphPad, San Diego, CA, USA), with *p* < 0.05 regarded as statistically significant.

### 2.7. Nuclear Effect Assessment

The effect of EO/FLC or thymol/FLC on *C. tropicalis* ATCC 200956 cells was assessed by staining with diamidino-2-phenylindole (DAPI) (Sigma-Aldrich, St. Louis, MO, USA). Cells at 4 × 10^5^ CFU/mL (2×) were treated with MICs and incubated at 35 °C for 4 h; then, yeasts were washed with PBS (1×), fixed in paraformaldehyde (4%) for 20 min, and permeabilized with Triton^TM^ X-100 (0.25%) (Sigma-Aldrich, St. Louis, MO, USA) for 60 min. Finally, cells were stained with 1 μg/mL of DAPI and incubated at room temperature in the dark for 30 min. The samples were collected by low-speed centrifugation, washed twice, and re-suspended in PBS (1×). Yeasts were visually analyzed under a fluorescence microscope at an emission/excitation wavelength (λ) of 341/452 nm, respectively. Untreated cells were used as a control.

### 2.8. Cell Cycle Assessment

To evaluate the effect of the EO/FLC and thymol/FLC on the cell cycle, *C. tropicalis* ATCC 200956 was grown on SDA at 35 °C for 24 h. Following this, a concentrated yeast solution was prepared in distilled water and kept at 25 °C for 2 h. The inoculum was adjusted at 4 × 10^5^ CFU/mL (2×) in RPMI + MOPS, treated with the MICs, and incubated at 35 °C for 4 h. Then, cells were fixed overnight with 70% cold ethanol and washed twice with 50 mM sodium citrate buffer before being treated with 20 µg/mL of RNAse A (Sigma-Aldrich, St. Louis, MO, USA) at 37 °C for 45 min. Two washes were done with a citrate buffer; the yeasts were resuspended in 200 µL of the same buffer. Finally, 100 µL of PI at 50 µg/mL was added to 30 µL of the yeast solution and incubated at 25 °C for 20 min in the dark. All experiments were performed in triplicate, and readings were carried out using an LSR Fortessa^TM^ flow cytometer (BD, Franklin Lakes, NJ, USA) and analyzed using FlowJo software, version 7.6. DNA content was estimated by measuring PI median fluorescence intensity (MFI); cell percentages with 1, 1.5, and 2 MFI as DNA content indicators were determined. Statistical analysis was performed by a one-way ANOVA test using the Prism 6.0 statistical program, with *p* < 0.05 regarded as statistically significant.

### 2.9. Morphology Assessment by Scanning Electron Microscopy

*Candida tropicalis* ATCC 200956 cells at 4 × 10^5^ UFC/mL (2×) obtained from an SDA culture at 35 °C for 24 h were incubated at 35 °C for 4 h with EO/FLC or thymol/FLC MICs. Cells treated with FLC, AMB, and caspofungin (CSF; Sigma-Aldrich, St. Louis, MO, USA) at 64, 8, and 4 µg/mL, respectively, were included as the positive control. Cells without treatment were used as the negative control. After treatments, the yeasts were fixed in glutaraldehyde (2.5%) overnight and then submerged in Sorensen’s phosphate buffer pH 7.0 for 12 h. Three washes were carried out with the same buffer, and one wash with distilled water at the end. The samples were fixed and dehydrated with increasing ethanol concentrations (50, 75, 95, and 100%). The samples were deposited on graphite tape and thinly coated with gold (Au) using a cold sputter coater (Denton Vacuum Desk IV, Beijing, China) and then observed using a High Vacuum Scanning Electron Microscope (SEM) (JEOL JSM 6490 LV Tokyo, Japan). A secondary electron detector was used to evaluate their morphology. Elemental analysis was determined using an INCA PentaFETx3 X-ray microprobe EDS (Oxford Instruments, Abingdon, UK).

### 2.10. Ultrastructure Assessment by Transmission Electron Microscopy

Ultrastructural evaluation by transmission electron microscopy (TEM) was performed on *C. tropicalis* ATCC 200956 cells in the exponential growth phase [4 × 10^5^ UFC/mL (2×)]. Cells were treated with EO/FLC or thymol/FLC MICs and incubated in agitation at 35 °C for 4 h, fixed in a sodium hydrogen phosphate buffer 0.1 M (pH 7.4), glutaraldehyde (2%), and paraformaldehyde (4%) at room temperature for 2 h, then washed three times with sodium hydrogen phosphate buffer and fixed with potassium permanganate (1%) at 4 °C for 1 h and tannic acid (0.15%) and uranyl acetate (2%) for 1 min and 1 h, respectively, at room temperature. The cells were dehydrated by incremental concentrations of ethanol (50, 75, 90, 95, and three times with 100%) at 4 °C for 10 min each. Infiltration was performed at room temperature by agitation using increasing concentrations of epoxy-resin (25, 50, 75, and 100%). Polymerization was performed at 60 °C for 48 h. Ultrathin sections of the samples (50 to 70 nm) were obtained with a Leica EM UC6 ultramicrotome and harvested on 100-mesh Formvar-coated copper grids and stained following standard procedures with 4% uranyl acetate and 2% lead citrate. Images were recorded at nominal magnifications of 15,000× to 67,000× with a CCD (Charged Coupled Device) FEI Ceta camera on a Tecnai 12 electron microscope (FEI) operated at 120 kV. The cell wall thickness was determined by measuring, in one or two points, at least 20 cells of each treatment with ImageJ software (version 1.52p). One-way analysis of variance (ANOVA) was carried out using the Prism 6.0 statistical program to identify significant differences between treated and control cells (*p* < 0.05 was regarded as statistically significant).

## 3. Results

Table 3 shows the GM-MIC values of FLC, EO, and thymol alone or in combination (EO/FLC and thymol/FLC) evaluated with two strains of *C. tropicalis* (azole-resistant and azole-susceptible). The susceptible and resistant FLC phenotypes were confirmed with the following MIC values: 2 and >64 µg/mL, respectively.

Additionally, the synergistic interaction between EO/FLC and thymol/FLC (FICI = 0.28) when the azole-resistant strain was tested resulted in a significant reduction in the individual MIC values: from >64 to 2 µg/mL (approximately 32-fold) in FLC and from 128 to 32 µg/mL (4-fold) in the EO and thymol. The thymol/FLC combination was synergistic with the azole-susceptible strain (FICI = 0.47). In this case, both FLC and thymol MIC values decreased from 2 to 0.60 µg/mL (3.3-fold) and from 256 to 42 µg/mL (6-fold), respectively. In contrast, the EO/FLC interaction was additive (FICI = 0.53). However, the reduction of the FLC MIC from 2 to 0.25 µg/mL was observed (8-fold).

**Figure 1:**
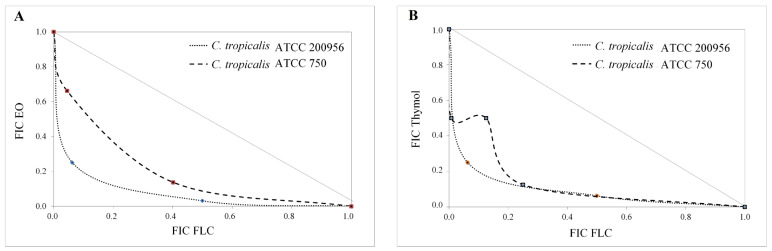
Isobologram of EO/FLC (**A**) and thymol/FLC (**B**) interaction against *C. tropicalis* strains. Points below the additivity line indicate a synergistic effect.

As mentioned in the experimental procedure, interactions between EO/FLC and thymol/FLC were represented by isobolograms (Figure 1). The straight line (additivity) indicates an indifferent effect, which means no interaction between the tested combinations was observed, while the concave lines below the additivity line indicate a synergistic effect of the combinations.

### 3.1. Lippia Origanoides EO or Thymol in Combination with FLC Reduce Mitochondrial Membrane Potential (∆ψm) but Do Not Affect the Plasmatic Membrane

**Figure 2:**
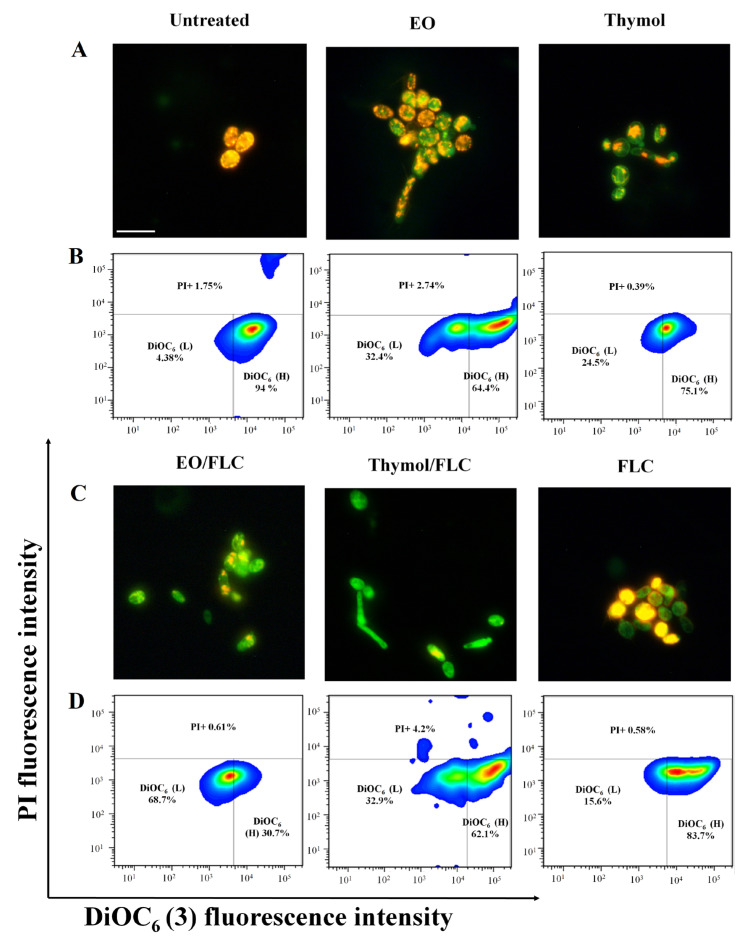
Effect of the different treatments on *C. tropicalis* cell mitochondrial membrane potential (∆ψm) and plasmatic membrane. (**A**,**C**) Photos of cells stained with JC-1 obtained by fluorescence microscopy. Green indicates monomers, and yellow indicates aggregates. (**B**,**D**) Representative plots of percentages of DiOC_6_(3) uptake cells (high (H) and low (L)). PI-positive cell percentages are shown above. Scale bar: 25 µm.

The effect on the mitochondrial membrane potential of EO, thymol, EO/FLC, or thymol/FLC was tested with JC-1 and DiOC_6_(3) fluorescent stains. In Figure 2A,C, the images show cells stained with JC-1 under fluorescence microscopy. Green fluorescence indicates the loss of mitochondrial membrane potential. Additionally, the JC-1 MFI was measured by cytometry, and the ratio of red/green was calculated. The results were as follows: EO (1.9), thymol (1.5), EO/FLC (2.4), thymol/FLC (2.0), FLC (2.0), negative control (3.9), and positive control (1.7). Lower values in the ratio of red/green indicate a greater effect in the mitochondrial membrane potential. Our results indicate that thymol was the treatment that caused the greatest loss of mitochondrial membrane potential, even above that observed in the positive control treated with AMB.

In Figure 2B,D, the percentages of high (H) and low (L) DiOC_6_(3) uptake cells are shown. Both JC-1 and DiOC_6_(3) results indicate mitochondrial depolarization. In addition, the percentages of positive cells for PI are indicated at the top of the panels of Figure 2B,D. The low percentage of cells that took up the PI evidenced that the evaluated treatments did not affect the integrity of the plasmatic membrane; 98% of the positive control cells captured the PI.

### 3.2. Thymol Used Alone and in Combination with FLC Increased Mitochondrial Superoxide Production

**Figure 3:**
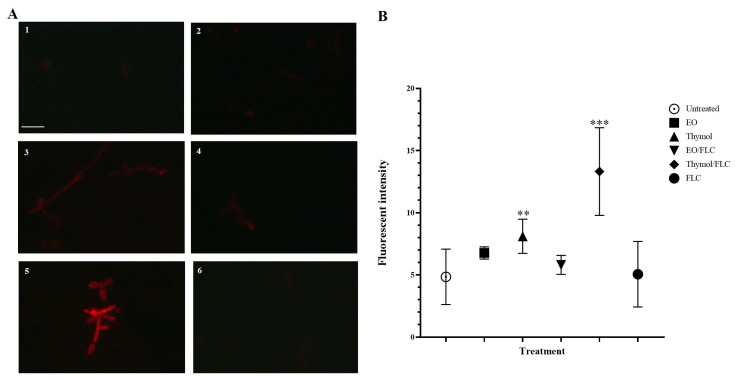
*Candida tropicalis* ATCC 200956 cells stained with MitoSOX^TM^ Red were observed under a fluorescence microscope. (**A1**) Untreated cells; (**A2**) cells treated with EO, (**A3**) thymol, (**A4**) EO/FLC, (**A5**) thymol/FLC, and (**A6**) FLC. (**B**) Mean fluorescence intensity values of cells with different treatments. ** *p* = 0.0008; *** *p* < 0.0001. Scale bar: 25 µm.

In Figure 3, microscopy fluorescence images and the quantification of MitoSOX^TM^ Red fluorescence intensity are shown. Superoxide anion (O_2_-) production was significant when thymol (*p =* 0.0008) and the thymol/FLC combination were added (*p* < 0.0001) (Figure 3B).

### 3.3. Lippia Origanoides EO or Thymol in Combination with FLC Causes Nuclear Alterations

**Figure 4:**
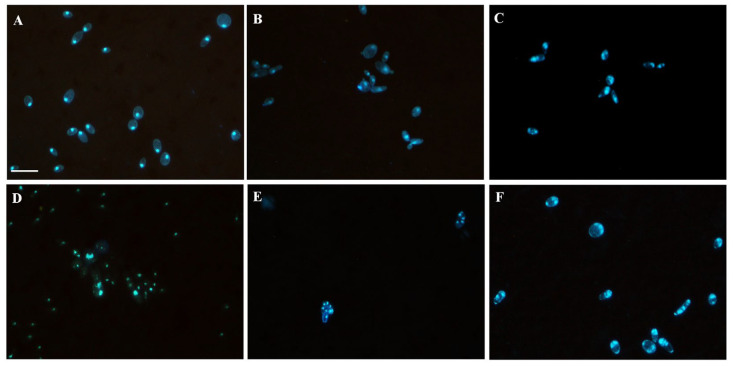
Fluorescence microscopy pictures depicting the changes on the nucleus of *C. tropicalis* ATCC 200956. (**A**) Untreated and treated cells with (**B**) EO, (**C**) thymol, (**D**) EO/FLC, (**E**) thymol/FLC, and (**F**) FLC. Scale bar: 25 µm.

Morphological changes in *C. tropicalis* ATCC 200956 nucleus were visualized in yeasts stained with DAPI and observed under a fluorescence microscope after EO, thymol, EO/FLC, thymol/FLC, or FLC treatments (Figure 4). Normal characteristics of the nuclei were observed in untreated cells (Figure 4A), while EO-treated yeast showed typical karyopyknosis, with condensed shrinkage cell nuclei (Figure 4B), which were more evident in the thymol-treated ones (Figure 4C). An unorganized and dispersed DNA-stained pattern was observed with FLC treatment (Figure 4D). Interestingly, in yeast treated with EO/FLC (Figure 4E) or thymol/FLC (Figure 4F), typical karyorrhexis, with small and compacted chromatin granules spreading into the cytoplasm, was observed.

### 3.4. Lippia origanoides EO or Thymol in Combination with FLC Causes Alterations in the Cell Cycle

**Figure 5:**
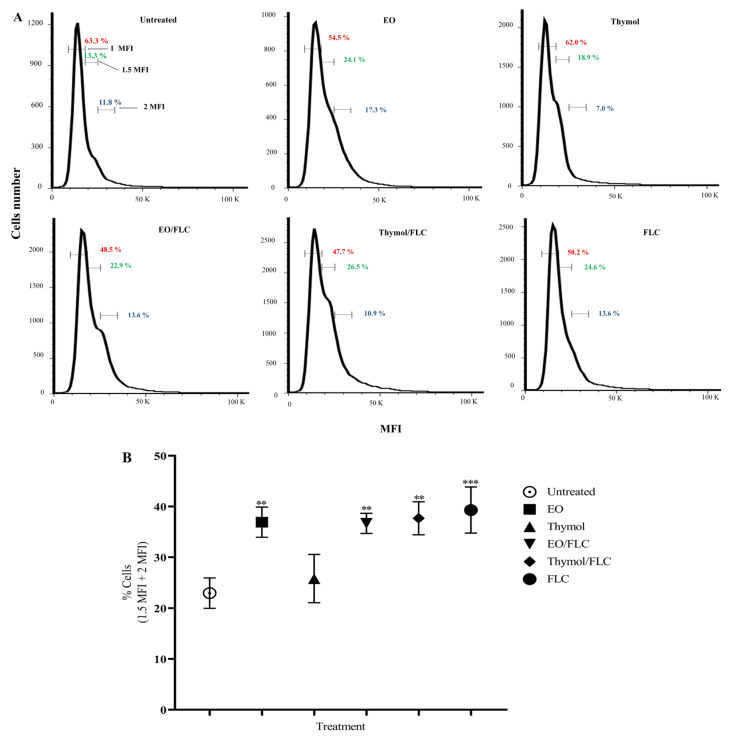
Effect of different treatments on the *C. tropicalis* ATCC 200956 cell cycle. (**A**) Representative plots of data indicating 1 MFI (red), 1.5 MFI (green), and 2 MFI (blue) cell percentage. (**B**) Average 1.5 MFI + 2 MFI cell percentage. MFI: Median fluorescence intensity. *p*-values: EO = 0.0056; EO/FLC = 0.0028; thymol/FLC = 0.0016; FLC = 0.0007. (** *p* < 0.01; ***p < 0.001).

The effect of EO, thymol, EO/FLC, and thymol/FLC on the *C*. *tropicalis* ATCC 200956 strain cell cycle was evaluated. Analysis of the DNA content estimated by the MFI of the PI showed a significant decrease in the percentage of 1 MFI cells after treatment with EO/FLC (48.5%; *p* = 0.039) and thymol/FLC (47.7%; *p* = 0.030) when compared to untreated cells (63.3%). In addition, a significant increase (*p* < 0.05) in polyploidy (1.5 MFI + 2 MFI) was observed after treatment with EO (41.4%), EO/FLC (36.5%), thymol/FLC (37.4%), and FLC (38.2%). DNA content in thymol-treated cells was similar to the control (25.9% and 25.1%, respectively). Moreover, all of the treatments except thymol increased the 1.5 MFI or 2 MFI cell percentage in comparison with untreated cells (Figure 5B).

### 3.5. Lippia origanoides EO or Thymol in Combination with FLC Cause Both Superficial and Ultrastructural Alterations

**Figure 6:**
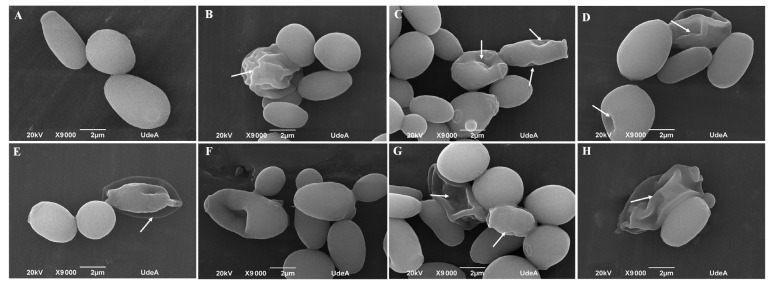
SEM images of *C. tropicalis* ATCC 200956: (**A**) untreated cells; (**B**) treated with EO, (**C**) thymol, (**D**) EO/FLC, (**E**) thymol/FLC, (**F**) FLC, (**G**) AMB, and (**H**) CSF. Arrows indicate alterations on the cell surface. AMB: amphotericin B; FLC: fluconazole; CSF: caspofungin; EO: essential oil.

SEM observations of untreated cells revealed a normal superficial appearance (Figure 6A). After exposure to the EO, thymol, EO/FLC, or thymol/FLC, aberrant morphologies, including wrinkles, shrinkages, and depression of the cell surface, were observed (Figure 6B–E respectively). Furthermore, a similar effect was observed in yeasts treated with FLC, AMB, and CSF (Figure 6F–H, respectively).

**Figure 7:**
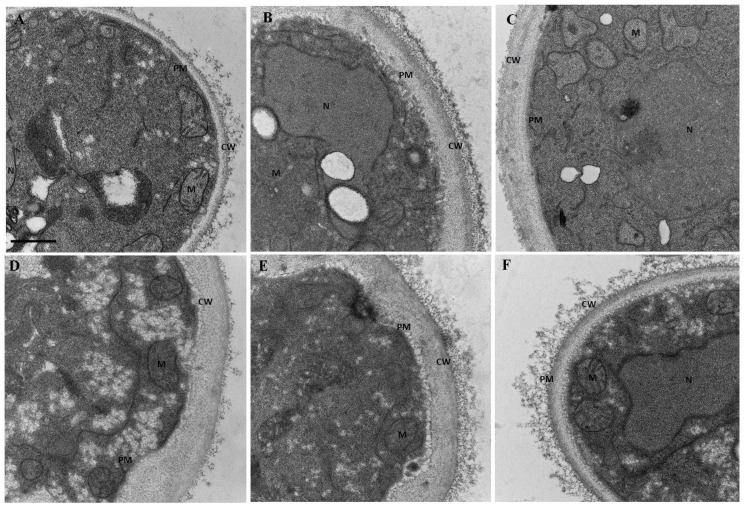
TEM micrographs of *C. tropicalis* ATCC 200956. (**A**) Untreated cells and cells treated with (**B**) EO, (**C**) thymol, (**D**) EO/FLC, (**E**) thymol/FLC, and (**F**) FLC. PM: plasmatic membrane; CW: cell wall; M: mitochondria; N: nucleus. Scale bar: 500 nm.

In Figure 7, micrographs obtained by TEM show the ultrastructural effect of treatments on *C. tropicalis* ATCC 200956 cells. Untreated or FLC-treated cells demonstrated small and round mitochondria with well-contrasted cristae and a homogeneously light matrix (Figure 7A,F). In contrast, yeasts treated with the EO or EO/FLC showed diffused cristae but meaningless changes in mitochondrial shape and size (Figure 7B,D). Conversely, morphology changes and loss of cristae in cells treated with thymol or thymol/FLC were observed (Figure 7C,E).

**Figure 8:**
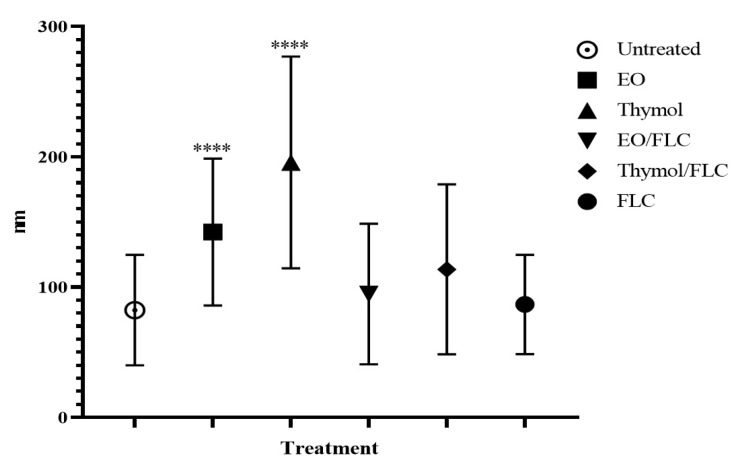
*Candida tropicalis* ATCC 200956 cell wall thickness measurement. **** *p* < 0.0001.

Changes in cell wall thickness were observed following each treatment. The cell wall width of at least of 20 cells was measured and is represented in Figure 8. All the treatments, except FLC, increased the cell wall width, but this increment was especially noteworthy with EO and thymol.

## 4. Discussion

The use of antifungal combinations and their amalgamation with other molecules is considered an effective strategy in reducing antifungal doses required for treatment, avoiding the development of resistance as well as reversing existing drug resistance, and inhibiting the formation or disintegration of biofilms [23,42]. The synergistic effect of EOs or terpenes in combination with FLC against both planktonic and biofilm growth of *C. albicans*, *C. tropicalis*, as well as the multidrug-resistant *C. auris* has already been demonstrated [23,25,52]. Our group previously demonstrated the anti-*Candia* activity of *L. origanoides* EOs and terpenes, both on planktonic and biofilm cultures [39,53]. Of note was the marked effect of the EOs and terpenes on resistant strains, including the *C. tropicalis* ATCC 200956 strain. This effect was greater than on the more susceptible strains [39]. In this current study, we investigated whether the interaction of *L. origanoides* EO or thymol potentiated FLC activity against the azole- and AMB-susceptible *C. tropicalis* ATCC 750 strain and the resistant *C. tropicalis* ATCC 200956 strain. For the latter strain, the resistance mechanisms, due to *ERG 11* and *ERG 3* gene mutations, the absence of ergosterol in the cell membrane, changes in the wall structure, and an activated antioxidant mechanism, were identified previously [11,54].

Table 2 and Figure 2 confirm the synergistic effect of EO/FLC and thymol/FLC combinations against *C. tropicalis* strains. The synergistic interaction with *C. tropicalis* ATCC 200956 led to a phenotypic shift (from resistant to susceptible), resulting in EO/FLC and thymol/FLC MIC values similar to those of the FLC-susceptible strain. The above results (Table 2 and Figure 2) show that the EO and thymol potentiated the FLC activity against the two strains, but this effect was more noticeable with the resistant strain.

Applying the Holetz et al. criteria for defining the activity of natural products [55], the activity of the EO/FLC and thymol/FLC mixtures against the resistant strain changed from moderate (GM-MIC = 128/64 µg/mL) to good (GM-MIC = 32/2 µg/mL), while in the susceptible strain, the EO/FLC changed from low (GM-MIC = 512/2 µg/mL) to moderate activity (GM-MIC = 181/0.25 µg/mL), and thymol/FLC from moderate (GM-MIC = 256/2 µg/mL) to good activity (GM-MIC = 42/0.60 µg/mL). These results strongly suggest that the EO and thymol potentiated the action of the FLC in the two strains, but more so with the resistant strain.

The use of strains with a known resistance mechanism to evaluate the antifungal activity of new molecules or phytocompounds can offer some insights not only to identify compounds with activity against resistant strains, but also to discover new targets or mechanisms of action.

A review by Bhattacharya et al. [21] described different mechanisms of synergistic action in EO–antifungal combinations. These comprise disruption of the cell wall structure and ergosterol biosynthesis pathway, pump/transporter activation in the cell membrane, cell membrane permeability alterations, intracellular leakage of cellular contents, inhibition of germ tube formation, fungal biofilm formation, and competition for a primary target. Furthermore, Zhang et al. proposed that the synergistic effect of *Melaleuca leucadendra* (L) EO and four antifungals from the azoles family against *C. albicans* was the result of mesosome-like structures forming around the cell membrane and in the cytoplasm. The EO altered the membrane permeability, thus facilitating antifungal cell penetration [45].

In this study, using TEM and SEM, we observed changes in external structure continuity and pores and an increase in the wall thickness after exposing cells to EO/FLC or thymol/FLC mixtures. Similar effects were observed with the antifungals (Figure 6 and Figure 7). The fungal plasmatic membrane and the cell wall have important cell functions as a stress response and allow the selective entry and exit of molecules. Damage to its structure affects normal physiological processes and leads to cell death [56]. Although we did not investigate the targets nor mechanisms of action that would allow us to explain the FLC-resistance phenotype reversal, it is reasonable to suggest that the *C. tropicalis* ATCC 200956 membrane composition changes (without ergosterol but with 14α-methylated accumulated sterols, as previously confirmed) [11], making the cell membrane more permeable to EO compounds and thymol. In addition to superficial morphological changes, the mitochondrial membrane potential of *C. tropicalis* ATCC 200956 cells was altered by all the treatments (Figure 2).

Several studies have demonstrated that changes in mitochondrial functions have an important role in the susceptibility to different antifungal molecules [28,57,58]. On the other hand, an increase in O_2-_ production was detected when cells were treated with thymol and thymol/FLC (Figure 3). Experiments carried out with different fungal models allowed for the detection of ROS production in mitochondria in response to antifungal compounds or to phytocompound mixtures. Reactive Oxygen Species affect cell viability due to cell membrane oxidation, affect proteins, lipids, and nucleic acids, and also activate proapoptotic signals [59,60,61]. For these reasons, mitochondria are considered a possible target for the development of new antifungal drugs [62].

Nuclear morphology and the cell cycle were affected by EO, thymol, FLC, and by their combinations (EO/FLC and thymol/FLC) (Figure 4 and Figure 5). The cell cycle analysis showed an increase in the amount of DNA (MFI 1.5 + MFI 2) (Figure 5). This may correspond to an increase in polypoidal cell states [63,64]. In yeasts, under optimal conditions, haploid and diploid cells as well as the number of chromosomes are usually stable; however, under stress conditions, it is possible to reach polypoidal states. It is known that tetraploid cells are arrested in the stationary phase, have unstable genomics, and lose chromosomes [63,65]. Additionally, polyploidy in yeasts is related to greater sensitivity to antifungal treatments, whose target is microtubule depolymerization or inhibition of DNA replication [63].

In a recent analysis of *C. tropicalis* ATCC 200956 and *C. tropicalis* ATCC 750 carried out by our group, we observed significant changes in gene expression in response to *L. origanoides* EO and thymol. The gene regulation implicated in energy metabolism, sterol synthesis, nucleosome assembly, mitotic spindle and microtubule organization, and transmembrane domains involved in wall lipid homeostasis fungal function were altered). These results concur with the findings in this study.

## 5. Conclusions

From the results shown in this paper, we are able to conclude firstly that *L. origanoides* EO or thymol interaction with FLC results in a synergistic antifungal effect in both azole- and AMB-resistant or -susceptible *C. tropicalis* strains. The effect was more notable with the resistant *C. tropicalis*, where the phenotype was reverted. Secondly, this synergistic effect also applied to their impact on fungal cell structures and important cell viability functions.

Further research is required to understand the mechanisms by which the synergism reverses the resistant *C. tropicalis* strain phenotype. This could shed light on new antifungal targets and mechanisms of action. Additionally, in vivo studies could be undertaken to confirm the findings of this work and evaluate the potential for the development of future antifungals.

## Figures and Tables

**Table 1:** Composition of the EO of *L. origanoides* thymol + *p*-cymene chemotype.

Plant Species and Chemotype	Collection Site	Voucher Number	Principal Compounds (Relative Amount, %)
*Lippia origanoides* (Thymol + *p*-cymene) chemotype	Bucaramanga—Santander, Colombia	UIS * herbarium 22039	Thymol (49.4), *p*-cymene (19.1), *γ*-terpinene (9.2), *β*-myrcene (5.2), *α*-terpinene (2.9), carvacrol (2.7), thymyl methyl ether (1.8), trans-*β*-caryophyllene (1.6), cis-*β*-ocimene (1.2), and limonene (0.9).

* UIS: Industrial University of Santander. The major compounds are indicated in bold.

**Table 2:** Combinations of the compounds evaluated.

Combination	FLC MIC (X)	Thymol or EO MIC (X)
1	0	8
2	0.5	4
3	1	2
4	2	1
5	4	0.5
6	8	0

Note: X = fold-number MIC values.

**Table 3:** Essential oil or thymol interaction with FLC on azole-resistant and azole-susceptible *C. tropicalis* strains.

Strain	Compound/ Combination	GM-MIC (µg/mL)	FICI	Interaction Type	No. Times ↓ MIC		
					FLC	EO	Thymol
***C. tropicalis*** ATCC 200956 *	FLC	>64	-	-	-	-	-
	EO	128	-	-	-	-	-
	Thymol	128	-	-	-	-	-
	EO/FLC	32/2	**0.28**	Synergistic	32	4	-
	Thymol/FLC	32/2	**0.28**	Synergistic	32	-	4
***C. tropicalis*** ATCC 750 **	FLC	2	-	-	-	-	-
	EO	512	-	-	-	-	-
	Thymol	256	-	-	-	-	-
	EO/FLC	181/0.25	0.53	Additive	8.0	2.8	-
	Thymol/FLC	42/0.60	**0.47**	Synergistic	3.3	-	6

GM-MIC: Geometric mean values of the minimal inhibitory concentration (MIC); FLC: Fluconazole; EO: essential oil; FIC: Fractional Inhibitory Concentration; FICI: FIC Index. Values in bold indicate a synergistic interaction (FICI ≤ 0.5); * azole-resistant; ** azole-susceptible. The arrow (↓) indicates a MIC decrease.

## Data Availability

The necessary data can be requested from the correspondence author.
